# The Svalbard Global Seed Vault: 10 Years—1 Million Samples

**DOI:** 10.1089/bio.2018.0025

**Published:** 2018-10-12

**Authors:** Åsmund Asdal, Luigi Guarino

**Affiliations:** ^1^ Nordic Genetic Resource Center, Alnarp, Sweden.; ^2^ Global Crop Diversity Trust, Bonn, Germany.

Genebanks worldwide conserve plant genetic resources with the purpose of making them available for improving food and nutritional security through research, plant breeding, and education. In gene banks, genetic diversity is conserved *ex situ* through different methods, as seeds, living plants, and plant tissues. Well-dried, vacuum-packed seeds of so-called orthodox species can stay viable at low temperatures for very long predictable periods of time. Thus, for many crops, seed storage is the optimal method for long-term *ex situ* conservation of genetic diversity in genebanks.

There may be some 1750 crop genebanks worldwide. About 130 of these hold >10,000 accessions each.^1^ Genebank collections are held by international, regional, and national institutes as well as by universities, breeding institutes, NGOs, private entities, and commercial companies. As an extra security measure for the conservation of valuable resources, international guidelines recommend safety duplication of genebank collections. All genebanks providing access to seeds for researchers and plant breeders are free to store security copies of these seeds at the Svalbard Global Seed Vault.

The Vault was opened in 2008. It has the capacity to store 4.5 million seed samples in three caverns dug into a mountain at the end of a 100 m tunnel. Svalbard is considered to be an ideal place for a facility of this kind. It has deep permafrost, securing seeds at low temperatures even if the artificial cooling fails and it combines being a remote and calm location with good infrastructure and the presence of public services.

Offering the world a safe place for seeds is in accordance with long standing Norwegian policies for supporting biodiversity conservation, and stakeholders all over the world trust that Norway will take good care of the seeds. The Vault is owned by the Norwegian government and managed through an agreement between the Norwegian Ministry for Agriculture and Food, the Global Crop Diversity Trust, and the Nordic Genetic Resource Centre (NordGen). The seeds in the Vault remain the property of the depositor, which can get the seeds back if their genetic material is lost or inaccessible from their own or from cooperating gene banks' repositories. Only the depositing institution can obtain access to the seeds it deposited in the Vault.

After 10 years of operation, the number of deposited seed samples has reached 1,060,987. The major part, about two-thirds, has been deposited by international agricultural research centers. Four of these have deposited >100,000 samples each: CIMMYT (International Maize and Wheat Improvement Center) in Mexico, IRRI (International Rice Research Institute) in The Philippines, ICRISAT (International Crop Research Institute for the Semi-Arid Tropics) in India, and ICARDA (International Institute for Agricultural Research in Dry Areas), which until recently had its genebank in Syria.

The largest depositors among national genebanks are the United States, Germany, Canada, Australia, The Netherlands, South Korea, and Switzerland. NordGen, the regional genebank of the Nordic countries, has also secured a significant part of its seed collection in the Vault. Genebanks in several developing countries have deposited seeds as well: Mali, Nigeria, Sudan, Uganda, Zambia, Burundi, North Korea, Myanmar, and Pakistan, among others.

The Vault now holds samples of about 5000 different species. Rice and wheat are represented by >150,000 seed samples each. Furthermore, 15 major cereal, vegetable, and forage crops are represented by >10,000 seed samples (Fig. 1). A review 5 years ago estimated that about a third of globally distinct samples of 156 crop genera with orthodox seeds are safety duplicated in the Vault, although some “minor” crops and countries such as China and India are underrepresented.^2^

**FIG. 1.**
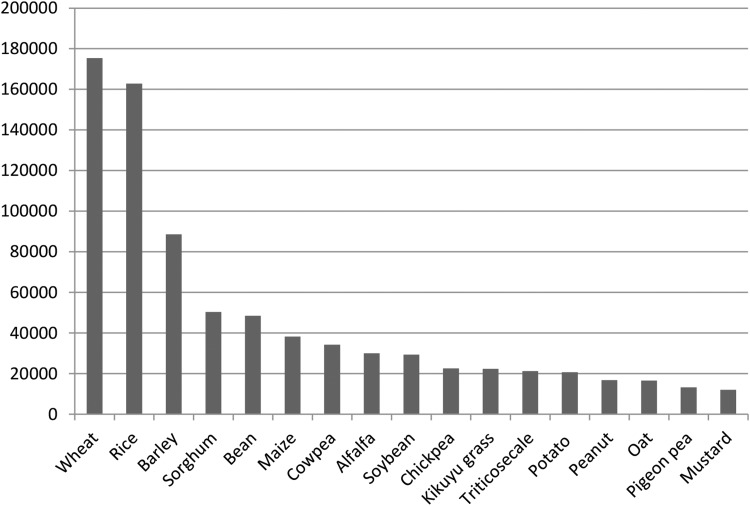
Number of seed samples stored in the Svalbard Global Seed Vault of the 10 best represented crops by March 2018. NordGen Seed Portal.^a^

So far, only one institute has requested seeds to be returned. This happened in the autumn 2015, when ICARDA, which until then had its headquarters in Aleppo, Syria, lost access to its genebank, and needed seeds from Svalbard to establish new functional genebanks at sites in Lebanon and Morocco. Seeds from the Vault have been shipped back on two occasions, and have been sown and multiplied at these sites. ICARDA has taken on the huge task of multiplying and redepositing major parts of its seed collections in the Vault in the shortest possible time. Since the spring of 2017, it has redeposited seeds in the Vault on three occasions.

The Svalbard Global Seed Vault is considered to be a vital part of the global system for conservation and use of plant genetic resources. This was reaffirmed at the last meeting of the Governing Body of the International Treaty on Plant Genetic Resources for Food and Agriculture (ITPGRFA) in November 2017, which also encouraged genebanks to make use of the Seed Vault in their strategies for the long-term security of important seed collections.^3^

The Vault partners invite genebanks to ship seeds to Svalbard on three or four regular opening occasions every year. Between 12 and 29 institutes have deposited seeds every year since 2008, for a total of 76. Many of these have deposited seeds several times, as part of a comprehensive program for securing major parts of their collections.^4^

After 10 years of operation, the Seed Vault is now undergoing improvements to make the storage even more secure toward future climate change scenarios. During melting periods, the Vault has experienced water leakage in the entrance tunnel, although not at all to the storage halls themselves. Despite concerns about climate change in the Arctic, Svalbard is still considered to be the optimal place for hosting the global backup for plant genetic diversity collections. The completely watertight entrance tunnel that will be built during 2018 and 2019 will further increase the security of deposited seeds for the future of agriculture and food production.

## Notes

^a^ (https://www.nordgen.org/sgsv/ 20180314).

## References

[1] FAO, 2010 The Second Report on The State of the World's Plant Genetic Resources for Food and Agriculture. www.fao.org/docrep/013/i1500e/i1500e.pdf (Accessed 315, 2018)

[2] Westengen OT, Jeppson S, Guarino L Global ex-situ crop diversity conservation and the Svalbard Global Seed Vault: Assessing the current status. PLoS One 2013;8:e64146 23671707 10.1371/journal.pone.0064146 PMC3650076

[3] The International Treaty on Plant Genetic Resources for Food and Agriculture, 2017. Resolution 12/2017. Cooperation with other International bodies and organizations. IT/GB-7/17/Res12

[4] NordGen, 2018 Svalbard Global Seed Vault Seed Portal, 20180314. https://www.nordgen.org/sgsv/ (Accessed 314, 2018)

